# Internet and written respiratory questionnaires yield equivalent results for adolescents

**DOI:** 10.1002/ppul.20576

**Published:** 2007-04

**Authors:** H Raat, RT Mangunkusumo, AD Mohangoo, EF Juniper, J Van Der Lei

**Affiliations:** 1 Department of Public Health, Erasmus MC-University Medical Centre RotterdamRotterdam, The Netherlands; 2 Department of Clinical Epidemiology and Biostatistics, McMaster University Medical CentreHamilton, Ontario, Canada; 3 Department of Medical Informatics, Erasmus MC-University Medical Centre RotterdamRotterdam, The Netherlands

**Keywords:** adolescents, allergy, asthma, international study of asthma and allergies in childhood (ISAAC) questionnaire, internet questionnaire, written questionnaire, validity

## Abstract

This study compared results from Internet and written questionnaires about respiratory symptoms in order to find out if both forms of the survey yielded the same answers. One thousand seventy-one students, ages 13 to 17, were asked to complete either an Internet or a written questionnaire. The demographic characteristics of the participants equalled those of the general Dutch adolescent population. Participants were randomly assigned to fill out an electronic or written questionnaire. In addition to eight items from the International Study of Asthma and Allergies in Childhood (ISAAC) questionnaire, two items on doctor visits (medical attention) regarding asthma or allergic disease during the past 12 months were included. The participation rate was 87%. The Internet version of the questionnaire showed fewer missing answers than the written version, but this was not statistically significant. The respiratory items did not show statistically significant score differences between the Internet and written modes of administration, and there was no visible trend for higher respectively lower scores by either mode of questionnaire administration. From these results, we conclude that respiratory questionnaires may be provided to adolescents electronically rather than on paper, since both approaches yielded equal results. To generalize these findings, we recommend repeated studies in other settings.

## INTRODUCTION

In addition to tests and clinical measures, validated respiratory self-report questionnaires constitute essential tools in respiratory disease epidemiology, screening procedures, monitoring patients' symptoms, and in clinical evaluation studies.^1^–^4^ The International Study of Asthma and Allergies in Childhood (ISAAC) questionnaire with eight items on wheezing and asthma is a widely used, validated respiratory symptoms questionnaire.^5^,^6^

Health questionnaires, such as the ISAAC questionnaire, are commonly distributed by means of paper forms, but researchers will increasingly be challenged to decide whether the original written version may be replaced by an electronic, respectively Internet version of the same instrument. Internet health questionnaires may be applied in a password-protected and secure environment and are accessible at the clinic, at home, or at school, providing practical advantages such as reducing paper work and eliminating manual data entry. In addition, Internet questionnaires allow dynamic question selection (e.g., skipping irrelevant questions) and data entry may be required before the respondent can proceed resulting in less missing data.^7^–^9^

However, mode of questionnaire administration (e.g., written questionnaire, face to face interview, telephone interview) may affect the participation rate, number of missing answers, psychometric properties, as well as the actual scores; it has been suggested that respondents may be hesitant to admit the presence of certain behaviors or events to another person in an oral interview; others hypothesized that patients may be eager reporting the presence of symptoms to a real interviewer (“pleasing”), compared to an anonymous questionnaire.^10^–^12^ Turner et al.^10^ showed that adolescents' reporting on sensitive topics such as drug use and unsafe sexual behaviors was higher (i.e., higher scores) by computer administered questionnaires (not by Internet) compared to written questionnaires, as adolescents apparently perceived the computer as providing more privacy than a paper procedure. Studies comparing the elicited scores of Internet and paper administration of health questionnaires, relatively few until now, showed varying results.^11^,^12^ ISAAC questionnaire score equivalence between Internet and paper administration has not yet been studied in adolescents.

In this randomized study among 13–17-year-olds, we assessed the results from an Internet and written version of the ISAAC questionnaire. Next to eight ISAAC items on wheezing and asthma, two items on the presence of doctor visits with regard to asthma or allergic disease during the past 12 months were included. We also evaluated the number of missing answers in both modes of administration.

We hypothesized that Internet and paper administration of the ISAAC provide the same scores since items on respiratory symptoms are, in general, not perceived as sensitive. Furthermore, we hypothesized that Internet administration of the ISAAC questionnaire using software checking for missing answers, results in fewer incomplete data compared to paper administration.

## MATERIALS AND METHODS

### Study Subjects

In 2003, an opportunity sample of seven secondary schools with various educational levels in the area of Vlaardingen (metropolitan area) and Harderwijk (rural area), The Netherlands was invited to participate in the study with their 3rd year classes (students 13–17 years of age). The invited schools joined the study with a total of 1,071 students (from 55 classes) who were invited to complete respiratory questionnaires. The students and their parents each received written information about the study several weeks before data collection; parents could refuse their children's participation, and participation by the students was voluntary.

### Questionnaire

The questionnaire included eight ISAAC items on wheezing and asthma 5,6 and two additional items asking whether medical attention had been sought during the last 12 months with regard to asthma or allergy. In addition, items on standard socio-demographic variables were included.

Based on the conventional written questionnaire, an Internet version was developed based on PHP 4.0.1, MySQL 3.22, and JavaScript 1.3. The Internet version automatically skipped items, not showing irrelevant questions to the respondent, when appropriate. The Internet version did not provide checks for consistency of answers. At the end of the questionnaire, the Internet version checked for missing answers and invited the respondent to complete the remaining unanswered items, if present; however, without proper “logout” the respondent could leave items unanswered.

### Randomization

Within each school class, each student was assigned a random number generated by SPSS. Students in a school class were rank ordered according to this number and half of the class (50% with highest ranking) was assigned to the Internet mode of questionnaire administration and the other half (50% with lowest ranking) to the written mode of questionnaire administration. Students completed the questionnaires at school in a classroom under supervision of a research assistant.

### Analysis

We considered differences with a *P*-value <0.05 as statistically significant differences. We used Chi-square tests to determine whether the socio-demographic items in the questionnaire showed statistically significant differences between the subgroup that completed the Internet questionnaire and the subgroup that completed the written version. The difference between the average numbers of missing answers per item of the respiratory questionnaire for each mode was assessed by the Mann– Whitney *U*-test. Given relatively few missing answers, missing data were recoded as “no” respectively “zero” before further analysis of the data; no corrections were made for inconsistent responses. The differences between responses in both subgroups to the dichotomous items of the respiratory questionnaire were assessed by Chi-square tests. Differences between responses to the ISAAC items “last 12 months number of attacks of wheezing” and “last 12 months how often sleep disturbed by wheezing,” were assessed by Mann–Whitney U-tests. The analyses were also performed in subgroups of the study population, that is, boys (n = 432), girls (n = 501), 13–14-year-olds (n = 399) and 15–17-year-olds (n = 534). Additionally, in the whole study population, we tested whether scores between the Internet and written modes of administration were significantly different from each other by multivariate models that included interactions with gender and age. All analyses were done using SPSS, Version 11.0.1. The medical ethical committee of the Erasmus MC-University Medical Centre Rotterdam approved the study.

## RESULTS

### Participants

Participation rate was 87.1%. The age range of participants was 13–17 years (mean 14.7; SD 0.68); 53.8% were female; 92.8% of the participants were born in the Netherlands; the majority attended lower secondary education. There were no statistically significant differences with regard to socio-demographic characteristics between the subgroups allocated to the Internet and written mode (Table 1). The demographic characteristics of the participants (age, gender, country of birth, and educational level) reflected those of the general Dutch adolescent population.^13^

**Table 1:** Characteristics of Participants in the Study Stratified for Internet and Written Mode of Questionnaire Administration (n = 933)

	Internet mode of administration (n = 458)		Written mode of administration (n = 475)		*P*-value
	n	% of participants	n	% of participants	Internet versus written mode
Age					
13–14 years	189	41.3%	210	44.2%	0.36^1^
15–17 years	269	58.7%	265	55.8%	
Gender					
Female	257	56.1%	244	51.4%	0.17^1^
Born in the Netherlands					
Yes	425	92.8%	441	92.8%	0.90^1^
Educational level of the school					
Lower secondary	271	59.1%	274	57.7%	0.88^2^
Intermediate secondary	85	18.6%	94	19.8%	
Higher secondary	102	22.3%	107	22.55	

1 Chi squared test df = 1.

2 Chi squared test df = 2.

### Missing Answers by Mode of Administration

On average, the number of missing answers per item of the respiratory questionnaire did not differ statistically significantly (*P* = 0.13) between modes of administration; the Internet mode showed on average 0.25% missing answers per item (range 0–0.66%) and the written mode on average 0.72% per item (range 0–1.68%).

### Score Differences by Mode of Administration

Table 2 shows the scores for 10 respiratory items by mode of questionnaire administration; the differences in absolute percentages range from 0.2% (item “Ever had wheezing or whistling” 16.9% vs. 16.7%; and item “Last 12 months wheezing limited speech” 1.7% vs. 1.5%) to 2.2% (item “Last 12 months sounded wheezy during/after exercise” 9.8% vs. 12.0%); in 4 of the 10 items, the Internet mode of administration showed a higher prevalence than the written mode of administration. However, none of the 10 respiratory items did show score differences that were statistically significant between the Internet and written modes of administration in the whole sample (Table 2) as well as in four subgroups (boys, girls, 13–14- year-olds and 15–17-year-olds; data not shown); the mode of questionnaire administration did not interact statistically significantly with gender and age.

**Table 2:** Scores by Mode of Administration (n = 933)

	Internet mode of administration (n = 458)	Written mode of administration (n = 475)	*P*-value
	% of participants	% of participants	Internet versus written mode
Respiratory disease and symptoms:			
Ever had wheezing or whistling			
Yes	16.9%	16.7%	0.94^1^
Ever had asthma			
Yes	10.7%	9.9%	0.69^1^
Last 12 months doctor visits regarding asthma			
Yes	4.8%	5.7%	0.55^1^
Last 12 months doctor visits regarding allergy			
Yes	13.8%	14.3%	0.81^1^
Last 12 months wheezing/whistling			
Yes	10.0%	10.5%	0.81^1^
Last 12 months number of attacks of wheezing			
1 to 3	6.8%	7.8%	0.35^2^
4 to 12	1.8%	1.3%	
4 or more	0.9%	1.3%	
Last 12 months how often sleep disturbed by wheezing			
Sometimes but less than one night per week	2.0%	1.7%	0.74^2^
One or more nights per week	0.2%	1.1%	
Last 12 months wheezing limited speech			
Yes	1.7%	1.5%	0.74^1^
Last 12 months sounded wheezy during/after exercise			
Yes	9.8%	12.0%	0.29^1^
Last 12 months dry cough at night (without a cold)			
Yes	16.6%	14.5%	0.38^1^

1 Chi squared test df = 1.

2 Mann–Whitney *U*-test.

### Power Analysis for Differences in Missing Answers and for Score Equivalence

We used the observed distributions of missing answers in the Internet group and written mode of administration group as an illustration of the power of the study. A study with 1,400 participants would have been required to reach adequate power (80%) to detect, at a significance level of *P* = 0.05, the difference between on average 0.25% missing answers per item in the Internet group compared to 0.72% in the written mode of administration group. In addition, with regard to differences in scores between the two groups, with the current data set, we could have found a significant score difference (*P* = 0.05) for “last 12 months wheezing/whistling” between 8.5% for the written mode of administration and 11.9% for the Internet group with a power of 80%.^14^

## DISCUSSION

In this first randomized adolescent study that compared Internet and written administration of the ISAAC asthma and wheezing questionnaire, we confirmed that an Internet version of such a health questionnaire was feasible. The study did not show statistically significant score differences between modes of administration in the case of this specific adolescent respiratory questionnaire; the results support comparative validity between paper and pencil and Internet administration of the ISAAC. The Internet mode of administration providing a control for not answered items, showed fewer missing answers than the written version, but this difference was not statistically significant. The results of this study are preliminary given the design and relatively limited power (see below).

Strengths of the study include the high participation rate and randomization to written and Internet administration. The reported prevalence of wheezing and asthma in this study was comparable to published rates in other childhood studies in the Netherlands andWestern Europe.^15^,^16^

There were some limitations to this study. It was performed in a school setting with relatively healthy adolescents. Questionnaires were completed under supervision, which may have enhanced participation and completion of the forms. Further evaluations should include unsupervised data collection such as on-line applications to be accessed from homes and clinics, and should include patient groups next to general population samples.

In this study, we only studied the paper and pencil version of the ISAAC and the Internet version of that questionnaire and did not evaluate the video-supported version of the ISAAC questionnaire.^5^

We applied a randomized parallel group design since a randomized crossover design may provide results that cannot be interpreted. A repeated measure with a health questionnaire within one person (without change in health condition) may cause small but statistically significant score differences that may equally be the result of a different mode of administration at the second measure (in the case of a cross-over design regarding an Internet vs. paper comparison), as simply by the repetition of the same measure (retest); for example, in a study with the Child Health Questionnaire, a generic adolescent health measure with 10 multi-item scales, we found significant score differences at the retest in 5 out of 10 scales without explanation.^17^,^18^ Unfortunately however, the current parallel group design does not allow evaluating whether changes in reported symptom status over time by a single person might be influenced by the mode of administration.

This study illustrated that it is feasible to have an Internet system check for missing answers and thereby reducing the number of missing answers, as was also found by Lonsdale et al.^19^ in a study with a psychological questionnaire among athletes. The power analyses showed that a future larger study might provide evidence of a statistical significant decrease in missing answers by Internet application of the ISAAC. However, since the written version of the questionnaire also resulted in relatively few missing answers, there was only a small benefit to gain in this study. Using an Internet questionnaire with the purpose of reducing missing answers may be advantageous in the case of other questionnaires, respectively study populations, where relatively many missing answers can be expected.

Turner et al.^10^ have suggested that computer questionnaires compared to written questionnaires might result in a higher reported prevalence when the items are of a sensitive nature. Ritter et al.^11^ found no score differences between Internet and written health questionnaires, while Mangunkusumo et al.^12^ reported two significant score differences between Internet and written questionnaires (satisfaction with appearance and having a sufficient number of friends). Given the absence of statistically significant score differences between administration modes, and since there was no visible trend for higher respectively lower scores by either mode of questionnaire administration, the results of the current study support the hypothesis that Internet and written administration of the ISAAC provide the same scores and that items on respiratory symptoms are not perceived as sensitive.

The differences between Internet and written versions of the ISAAC as shown in this study were generally small. However, it is arbitrary what constitutes a meaningful prevalence difference between the two modes of ISAAC administration and this may depend on the aims of a study. Although the sample size, with 933 adolescents participating in our study, was relatively large from a practical perspective, we recommend larger studies in the future to establish whether ISAAC score differences such as for the item “Last 12 months sounded wheezy during/after exercise” in our study (9.8% vs. 12.0%), are caused by chance or by administration mode effects.

From the current data, we conclude that respiratory questionnaires may be provided to adolescents electronically rather than on paper, since both approaches yielded equal results. To generalize these findings, we recommend repeated studies, preferably with larger samples, in other settings.
